# The half-life of maternal transplacental antibodies against diphtheria, tetanus, and pertussis in infants: an individual participant data meta-analysis

**DOI:** 10.1016/j.vaccine.2021.12.007

**Published:** 2021-12-21

**Authors:** Blanché Oguti, Asad Ali, Nick Andrews, Daan Barug, Duc Anh Dang, Scott A Halperin, Ha Thi Thu Hoang, Beth Holder, Beate Kampmann, Abdul M Kazi, Joanne M. Langley, Elke Leuridan, Naomi Madavan, Kirsten Maertens, Herberth Maldonado, Elizabeth Miller, Flor M Munoz-Rivas, Saad B. Omer, Andrew J. Pollard, Thomas F. Rice, Nynke Rots, Maria E. Sundaram, Nasamon Wanlapakorn, Merryn Voysey

**Affiliations:** a Oxford Vaccine Group, Department of Paediatrics, University of Oxford, United Kingdom; b Aga Khan University, Karachi, Pakistan; c Public Health England, United Kingdom; d Centre for Infectious Disease Control, National Institute for Public Health and the Environment, the Netherlands; e National Institute of Hygiene and Epidemiology, Vietnam; f Canadian Center for Vaccinology, Dalhousie University, Canada; g Department of Metabolism, Digestion and Reproduction, Imperial College London, United Kingdom; h London School of Hygiene and Tropical Medicine, United Kingdom; i Centre for the Evaluation of Vaccination, Vaccine & Infectious Diseases Institute, University of Antwerp, Belgium; j University of Exeter, United Kingdom; k Universidad del Valle de Guatemala, Guatemala; l Baylor College of Medicine, USA; m Yale Institute for Global Health, USA; n Center for Clinical Epidemiology and Population Health, Marshfield Clinic Research Institute, Marshfield, USA; o Center of Excellence in Clinical Virology, Department of Pediatrics, Faculty of Medicine, Chulalongkorn University, Thailand

## Abstract

**Aim:**

There are few reliable estimates of the half-lives of maternal antibodies to the antigens found in the primary series vaccines. We aimed to calculate the half-lives of passively acquired diphtheria, tetanus and pertussis (DTP) antibodies in infants. We aimed to determine whether decay rates varied according to country, maternal age, gestational age, birthweight, World Bank income classifications, or vaccine received by the mother during pregnancy.

**Methods:**

De-identified data from infants born to women taking part in 10 studies, in 9 countries (UK, Belgium, Thailand, Vietnam, Canada, Pakistan, USA, Guatemala and the Netherlands) were combined in an individual participant data meta-analysis. Blood samples were taken at two timepoints before any DTP-containing vaccines were received by the infant: at birth and at 2-months of age. Decay rates for each antigen were log_2_-transformed and a mixed effects model was applied. Half-lives were calculated by taking the reciprocal of the absolute value of the mean decay rates.

**Results:**

Data from 1426 mother-infant pairs were included in the analysis. The half-lives of the 6 antigen-specific maternal antibodies of interest were similar, with point estimates ranging from 28.7 (95% CI: 24.4 – 35) days for tetanus toxoid antibodies to 35.1 (95% CI: 30.7 – 41.1) days for pertactin antibodies. The decay of maternal antibodies did not significantly differ by maternal age, gestational age, birthweight, maternal vaccination status or type of vaccine administered.

**Conclusion:**

Maternal antibodies decay at different rates for the different antigens; however, the magnitude of the difference is small. Decay rates are not modified by key demographic or vaccine characteristics.

## 1. Introduction

Neonates have largely naïve immune systems that leave them vulnerable to infectious diseases in early life. They are reliant on passive immunity gained from the pathogen-specific maternal antibodies transferred from their mothers, during pregnancy and lactation [1]. Maternal IgA antibodies are transferred postnatally in breast milk and predominantly act locally in the gastrointestinal tract. Maternal IgG antibodies are transferred transplacentally *in utero* and enter the fetal circulation, where they can modulate the infant immune response to pathogens [2,3].

Active transfer of maternal IgG antibodies to the fetus occurs across the placenta, mediated by the neonatal Fc receptors (FcRn) expressed on the surface of the syncytiotrophoblast, which invaginates to form the internal surface of the endosome, during transcytosis [4]. This process commences in early pregnancy [5] and increases in efficiency, as gestation progresses and FcRn expression increases [6]. Although FcRn binds to all IgG subclasses, the antibodies have differential transfer rates [7], based on multiple factors including Fc glycosylation, antigen specificity and functional potential [8]. A recent review suggested recent prenatal vaccination positively correlated with maternal antibody titres in the infant, as did good maternal nutrition, higher gestational age and male infant gender, to a lesser degree [9,10]. Principally, the serum concentration of antibody in the mother is considered the strongest predictor of the level of neonatal antibodies followed by gestational age [9,10].

Neonates are born with a limited quantity of transplacentally-derived IgG antibodies, which are mostly metabolized within the first six to twelve months of life [11]. The initial titre and decay rate of pathogen-specific maternal antibodies determines how long passive immunity will last [10]. If the concentration of maternal antibodies falls below the level of protection before the infant has completed their vaccination course, then the infant becomes susceptible to infection [2].

Maternal immunisation programs have been introduced in many countries, designed to protect the infant through boosting maternal antibody levels, hence augmenting neonatal immunity [12]. These have been successful in reducing infant mortality, most markedly with maternal pertussis immunisation; a UK study estimated maternal pertussis vaccine effectiveness against infant mortality to be 95%, three years after the national program was implemented [13].

The presence of transplacental maternal IgG antibodies at the time of infant vaccination can have an inhibitory effect on antibody generation by the infant’s immune system [11]. The exact mechanism and clinical significance of this blunting effect remains unclear, though many plausible hypotheses have been documented [11,14,15], including antigen neutralization, epitope masking, active inhibition of infant B cell activation, Fc-dependent phagocytosis of maternal antibody-coated antigens and affecting germinal B cell differentiation [15]. Since transplacental maternal antibodies decay over time, the concentration of maternal antibody present in the infant at the time they commence their infant series of vaccinations depends on the initial antibody level at the time of birth and the timing of the first dose of vaccine for the infant.

We previously modelled the interaction between the inhibitory effect of maternal antibodies on infant immune responses, and the timing of the first dose of infant vaccination for diphtheria, tetanus and pertussis antibodies [16]. This analysis required estimates of the rate of decay of maternal antibodies that were obtained from the published literature. Many published half-lives of passively acquired antibodies were from studies conducted some decades ago, and prior to introduction of maternal pertussis vaccination programmes. Estimates varied and generally came from studies with small sample sizes.

Due to the increasing focus on maternal prenatal immunisation as a means of protecting young infants against disease [17,18], it is necessary to have accurate and precise estimates of maternal antibody half-lives that can be used in mathematical models of infectious disease dynamics, and in planning of routine immunisation programmes that incorporate maternal prenatal vaccination. We combined data from multiple studies to estimate the rate of decay of maternal antibodies to diphtheria, tetanus and pertussis antigens in infants, and to explore factors that might influence the rate of decay, focussing on maternal age, gestational age, birthweight, sex of the infant and type of vaccine given.

## 2. Methods

We combined data in an individual participant *meta*-analysis from studies in which blood samples were obtained from infants at birth, and at one other time point prior to receipt of their first dose of infant diphtheria, tetanus, and pertussis-containing (DTP) vaccine. Studies were those from known collaborators with available data and not determined through systematic review, as the research question for the meta-analysis was different to the research questions for the individual studies. The research question in the meta-analysis was observational and non-interventional (decay rate of maternally transferred antibodies) whereas the individual studies predominantly tested vaccine interventions during pregnancy. We applied no restriction on the research question in the individual studies. We included both observational studies and randomised controlled trials with no restriction on whether vaccines were administered in the individual studies. Only studies in which the investigators measured IgG antibodies against diphtheria toxoid (DT), tetanus toxoid (TT), or pertussis antigens (fimbriae (FIM 2/3), pertussis toxin (PT), filamentous haemagglutinin, pertactin) were included. Results from infants who had antibody levels below the limit of detection of the assay at birth were excluded as half-lives could not be calculated, as were data from infants whose antibody levels increased over time as these indicated likely infections. Missing antibody data was not imputed therefore only infants with both blood samples available were included.

The vaccines used for maternal prenatal immunisation in these studies (where applicable) were against diphtheria, tetanus or pertussis, in different combinations; Td – tetanus toxoid and diphtheria toxoid vaccine; Tdap – tetanus, diphtheria, acellular pertussis (Boostrix© and Boostrix-IPV© [GlaxoSmithKline], Repevax© [Sanofi Pasteur] or Adacel© [Sanofi Pasteur] (Table S1). In studies with a randomised control group, these mothers received tetanus toxoid vaccine alone, Td, Tdap vaccine after giving birth, or no vaccine. Pregnant women who did not receive any prenatal vaccines, formed the entire cohort of one observational study in Pakistan.

This work was supported by the IMmunising PRegnant women and INfants neTwork (IMPRINT) funded by the GCRF Networks in Vaccines Research and Development which was co-funded by the MRC and BBSRC. This UK funded award is part of the EDCTP2 programme supported by the European Union. Studies for inclusion in the analysis were identified through the IMPRINT network collaborators.

## 3. Analysis

For each infant and each antigen, the antibody decay rate was calculated as the change in log_2_-antibody titre between the first and second blood samples, divided by the number of days between the visits. A one unit decrease on the log_2_ scale is a reduction by half, and thus log base 2 was used rather than a natural log as the sensible choice for the analysis of half-lives. The decay rate (the change per unit of time) rather than the half-life, the time it takes for half the quantity of antibody to be metabolized, was the unit of measure used in all analyses.

To calculate the mean decay rate for each antigen, a one-stage approach was used: a mixed effects model was fitted with study-specific random intercepts and no fixed effects. The mean decay rate was the intercept from the model and its 95% confidence interval, and this was converted into a half-life with 95% CI by taking the reciprocal of the absolute value of the intercept, and the reciprocal of its confidence limits. P-values were adjusted using the Bonferroni method [19] to account for multiple testing due to the analysis of six antigens.

To calculate whether decay rates differed by maternal factors, infant factors, or by vaccine type, the factor of interest was added into the model as a fixed effect. For continuous variables such as birthweight and maternal age, the variable was first assessed using a loess curve, to confirm whether the relationship between the antibody decay and the continuous predictor was a linear relationship. The continuous predictor was then added into the model. Continuous variables were also categorised into three groups to enable clearer presentation of the output, however the p-value from the continuous model was retained. The conclusions from continuous and categorised variables were the same.

These analyses were conducted using SAS version 9.4 and Stata version 16.0. Plots were generated using the cowplot and ggplot2 packages in R version 3.6.1

## 4. Results

Data from 1426 infant-mother pairs were available for these analyses and included ten studies from nine countries: Belgium, Canada, Guatemala, The Netherlands, Pakistan, Thailand, The United Kingdom (UK) (n = 2), the United States (USA) and Vietnam. Table 1 presents the characteristics of the studies listed by country cohort, with pooled descriptive data. In studies in which prenatal vaccines were administered, the median gestational age of the infants at the time of maternal vaccination was 30.9 (IQR 28 – 34) weeks. The median maternal age was 28.7 years old, with the youngest aged 16 and the eldest aged 44. The age of infants at the time of the second blood sample ranged between 2.9 and 11.9 weeks, with a median age of 8.7 weeks old. The median birthweight of the infants was 3180 (IQR 2825–3500) grams.

**Fig. 1.**
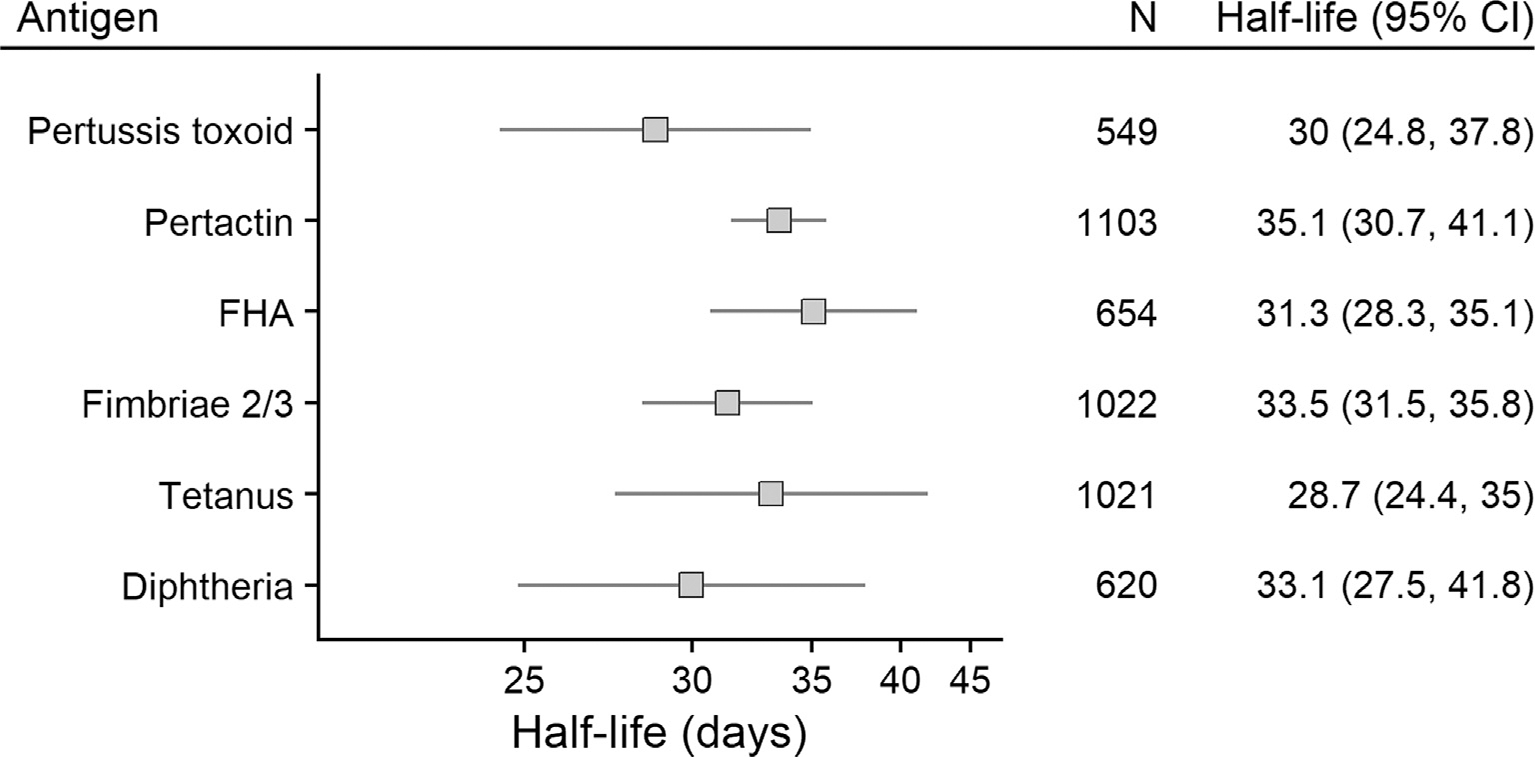
Half-lives of transplacental maternal antibody. FHA: Filamentous haemagglutinin.

The half-life of transplacental maternal antibodies ranged from 28.7 (95% CI: 24.4 – 35.0) days for tetanus-specific maternal antibodies to 35.1 (95% CI: 30.7 – 41.1) days for pertactin-specific maternal antibodies (Fig. 1).

**Fig. 2.**
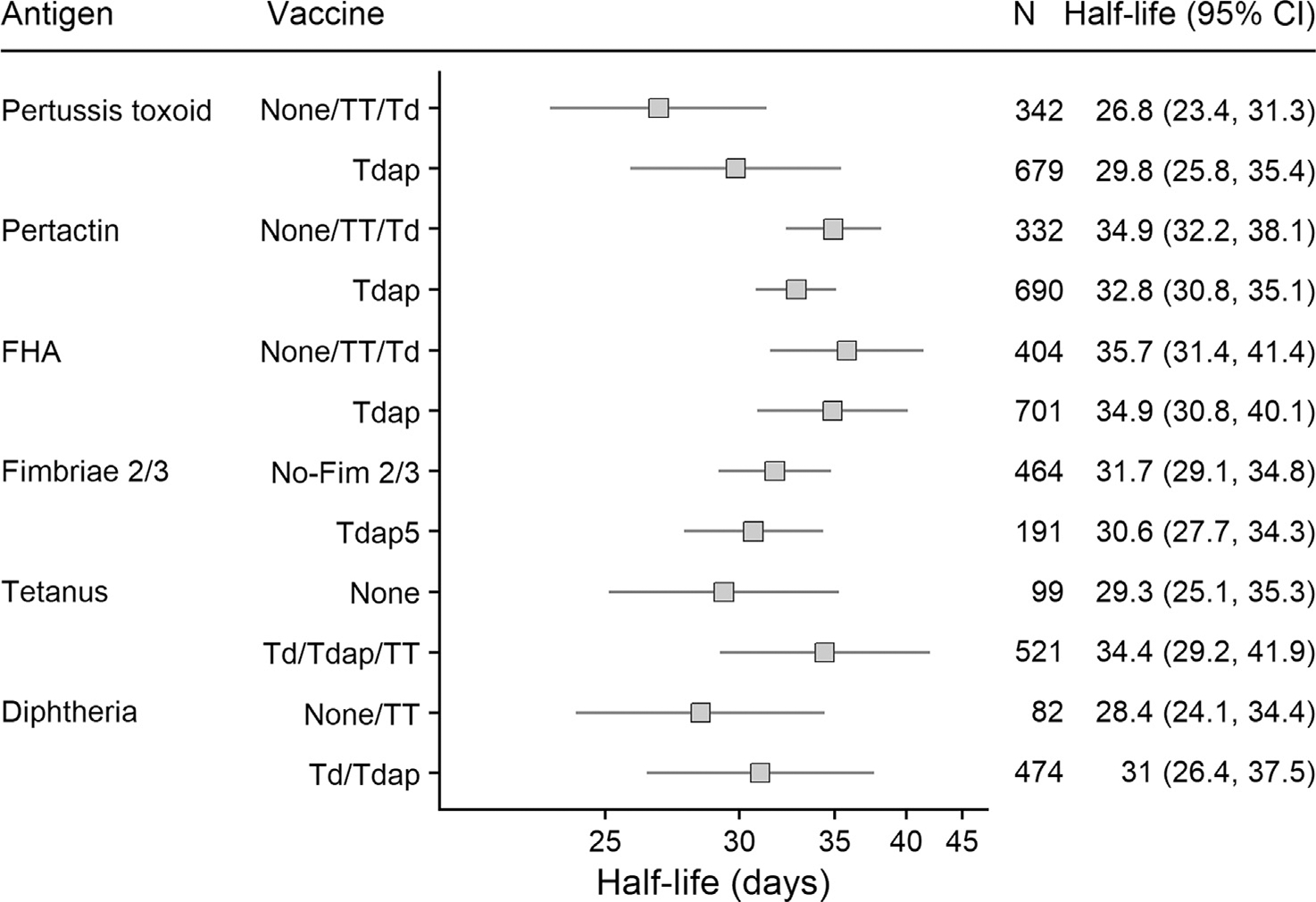
Half-lives of transplacental antibody in infants from mothers who did or did not receive a vaccine containing the same antigen during pregnancy. FHA: Filamentous haemagglutinin; TT: Tetanus toxoid; Td: Tetanus and diphtheria vaccine; Tdap: tetanus, diphtheria, and acellular pertussis vaccine. Adjusted p values for interaction terms: PT:0.002; PRN: 0.59 FHA:0.99, Fim 2/3:0.99, Tetanus:0.011, Diphtheria: 0.48.

Decay rates in infants were compared between mothers who had received a prenatal vaccination containing the antigen of interest or not (Fig. 2). The p-values for the difference between vaccination groups were significant for pertussis and tetanus toxoid only. An analysis exploring the half-life of maternal transplacental pertussis toxin antibody in infants from mothers who received acellular pertussis vaccines during pregnancy, showed that there was no significant difference by the type of vaccine used in the studies (Figure S1).

**Fig. 3.**
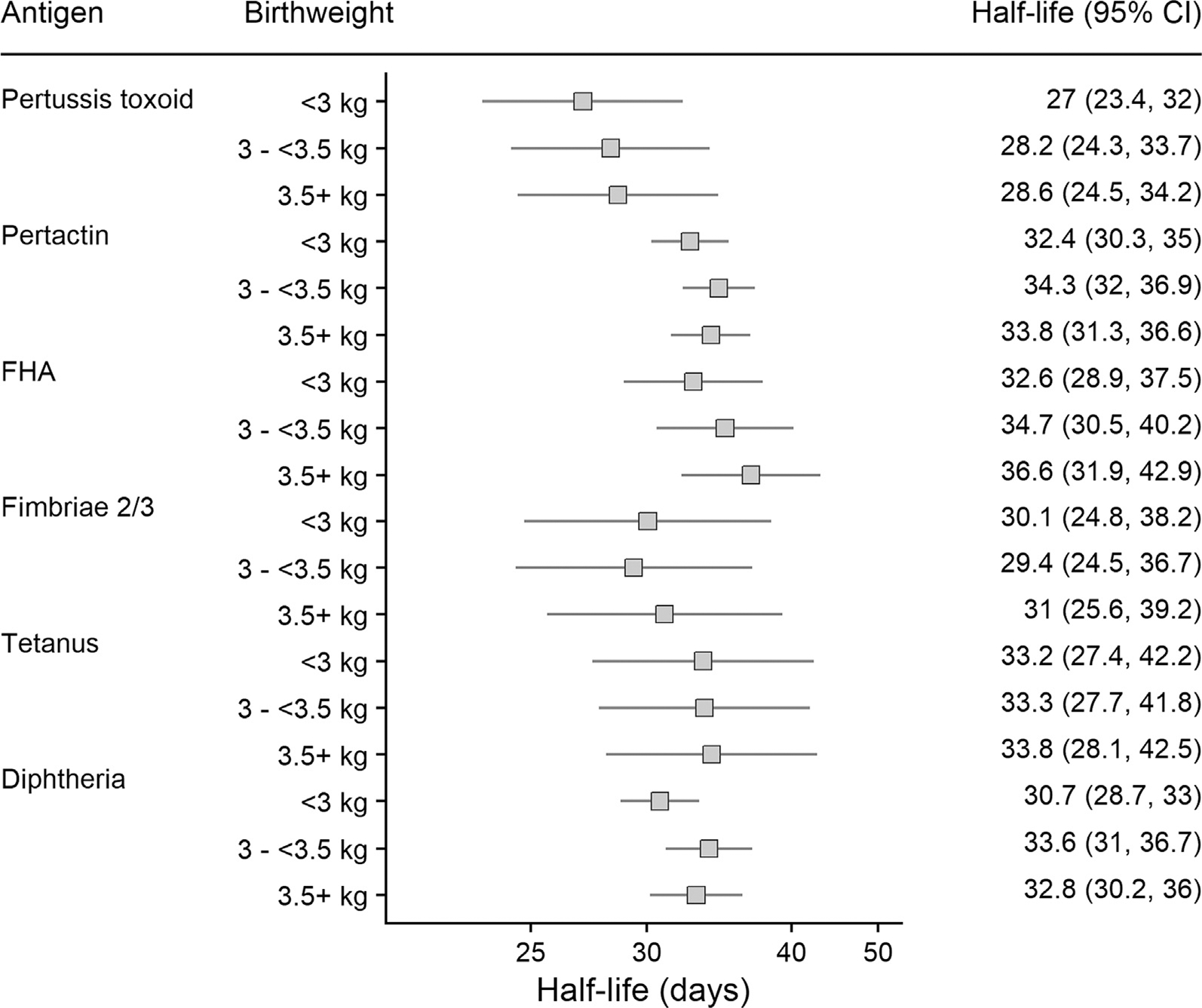
Half-lives of maternal transplacental antibody in infants by birthweight. FHA: Filamentous haemagglutinin.

**Fig. 4.**
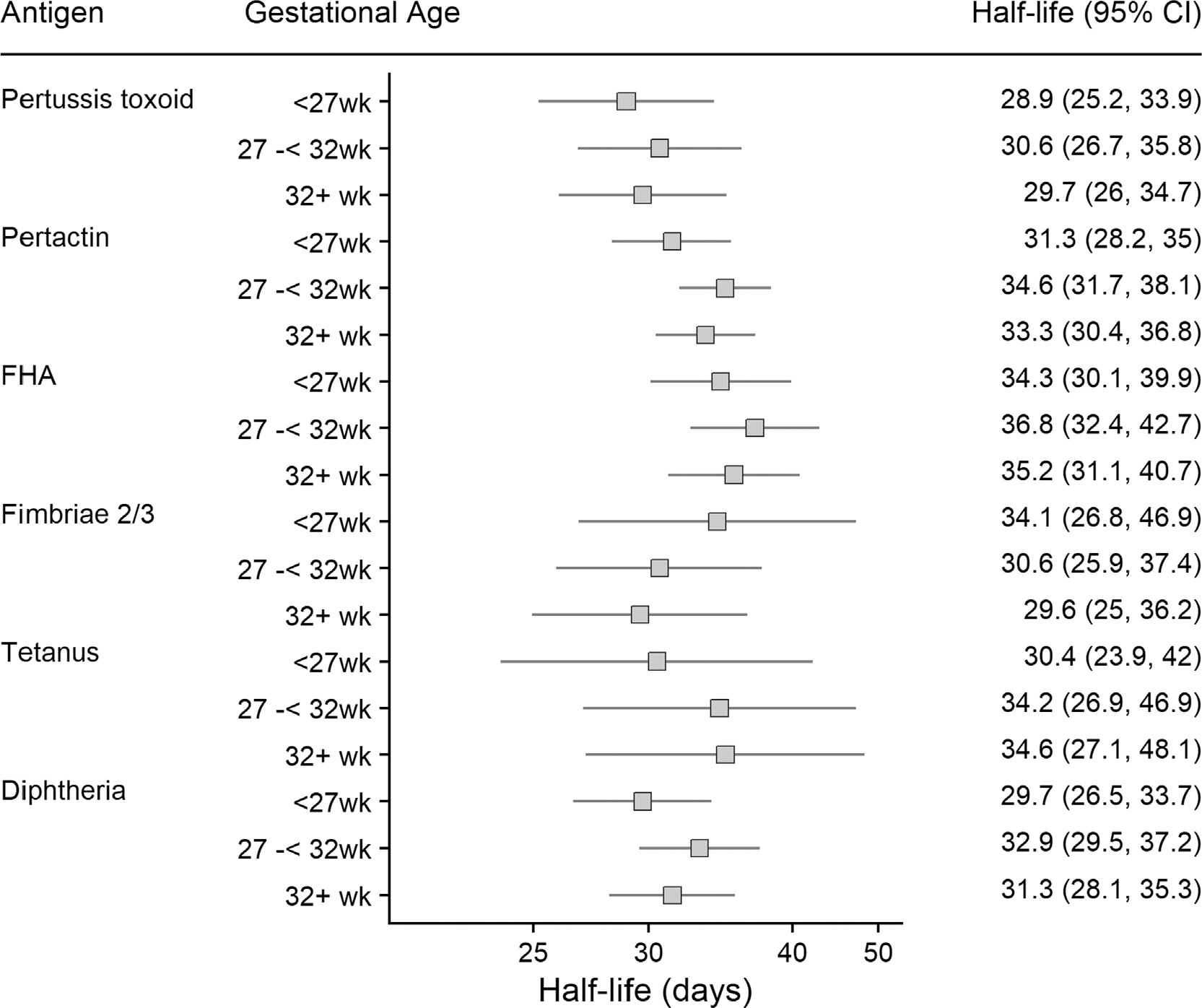
Half-lives of maternal transplacental antibody in infants by gestational age at vaccination, FHA: Filamentous haemagglutinin.

**Fig. 5.**
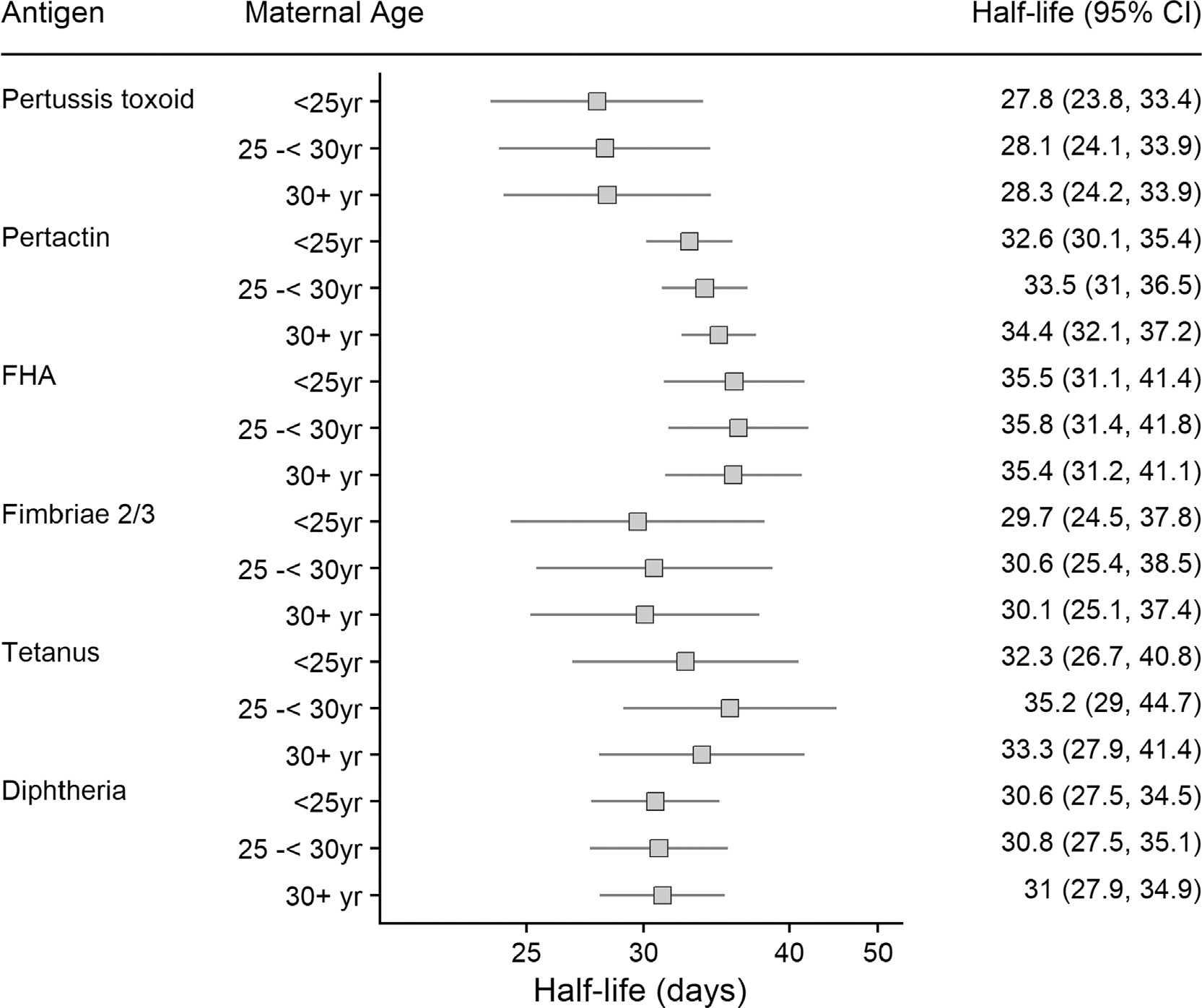
Half-lives of maternal transplacental antibody in infants by maternal age. FHA: Filamentous haemagglutinin.

The decay rate of maternal antibody did not vary by infant birthweight (Fig. 3), gestational age at vaccination (Fig. 4), maternal age (Fig. 5) nor by the World Bank income category of the country setting of the study (Figure S2).

Figure S3 displays all the individual infant data, of paired transplacental antibody titres, grouped by country cohort and antigen of interest.

## 5. Discussion

This study provides robust estimates of the half-life of transplacentally-transferred diphtheria, tetanus and pertussis antibodies in a large number of infants across multiple countries and shows that decay rates are consistent across different antigens and different epidemiological factors.

Combining data from 1426 infants and their mothers allowed for the exploration of several factors that have been postulated to modify the rate of transplacental antibody transfer and the kinetics of their decay. Adequate consideration of the effect of maternal transplacental antibody on infant antibody responses to vaccination is crucial for the planning of infant immunisation programmes in the context of expanding maternal vaccination programmes.

The overall estimated half-lives of transplacental maternal antibodies against diphtheria, tetanus, fimbriae, pertussis toxoid, filamentous haemagglutinin and pertactin antigens varied; however, the magnitude of the differences was no more than one week. Maternal antibody half-life estimates ranged between 28.7 and 35.1 days. This is in keeping with previous studies that have estimated the half-lives of FHA-, PT- and DT-specific maternal antibodies to be 40, 36 [29], and 35 days [30], respectively and slightly higher for anti-TT antibodies at 49 days [31,32]. This would suggest that by the time infants receive their first DTP-containing vaccines at 8 weeks, according to many national immunisation schedules, a quarter of their initial maternal antibodies will still be present. Although it is unclear what the clinical significance of this might be, it has been shown that maternal antibodies can inhibit infant immune responses to vaccination, even at low titres [33].

There is good evidence that blunting of the infant immune response to vaccination by the presence of maternal antibody, occurs for most antigens [16]. The blunting effect is greater with higher titres of maternal antibody circulating in the infant at the time [34]. Our analyses did not reveal any factors that modify the rate of maternal antibody decline, for any of the vaccine antigens of interest. This is in keeping with findings from the limited literature available [32].

In our study, there was no difference in the decay rates of infant vaccine-induced maternal antibodies compared to maternal anti-bodies in infants born to mothers who were not vaccinated during pregnancy. Other studies have found that infants from vaccinated mothers have higher initial titres of maternal antibodies compared to infants from unvaccinated mothers [10], therefore, if the decay rates are similar, these infants still have higher titres by receipt of their first DTP-containing vaccination. These findings support the theory that infants exposed to maternal vaccinations may be at higher risk of immunological blunting when they receive both primary and booster vaccinations [16,35]. Informed by comparable research conclusions, a two-tier system of infant immunisation has been introduced in the Netherlands; infants are either receiving their first Tdap vaccination at the established 3- month timepoint or receiving an additional dose at 2-months, before their 3-month vaccination, depending on whether their mother was vaccinated during pregnancy [36].

Multi-component vaccines have varying compositions with different levels of each antigen, depending on the vaccine developer. There was no evidence that the decay rate of pertussis antibodies was influenced by type of Tdap vaccine used for maternal immunisation. This is an important finding, as country-level decisions on the timing of maternal and infant pertussis vaccinations would be more complicated if the composition of the vaccine brands available produced significantly heterogeneous antibody decay rates. Since the quantity of pertussis antigens contained in each Tdap vaccine varied, one might expect the starting levels of vaccine-induced antibody to vary. The significance of this is difficult to characterize, as there are no quantifiable correlates of protection against pertussis to help navigate the space between immunological blunting and vulnerability to life-threatening infection.

In our study, infant gestational age at the time of maternal vaccination and infant birthweight made no significant difference to the decay rates of the transplacental maternal antibodies after birth. The transfer of IgG across the placenta is a saturable process; the quantity of maternal antibody that can be transferred to the fetus is limited by the amount of FcRn present in placental syncytiotrophoblast [4,11]. Several studies have found that greater infant gestational age relates to a higher concentration of maternal antibodies in the infant at birth [37]. It has been speculated that the increase in placental mass is responsible for the peak in the rate of transplacental antibody transfer in the last month of pregnancy [10]. Our data support established theories that higher antibody titres persist due to the kinetics of antibody decline [11]. Given that gestational age and birthweight [6] influence the concentration of maternal antibody in the infant at birth, without modifying decay rates, these factors need to be considered when determining the best time to vaccinate pre-term and low-weight babies. Pou et al. also found that maternal IgG concentrations were higher in term infants compared to preterm children, with comparable decay rates, which lead to longer IgG half-lives in term infants [9].

The decay rates of maternal antibodies to diphtheria, tetanus and acellular pertussis antigens in infants were not affected by maternal age. There are studies that have shown that maternal age influences the rate of transplacental antibody transfer; however, this does not translate to an impact on decay rates [37].

A strength of our analysis is the availability of paired samples in the same infant, allowing for analysis of within-person change in antibody levels. The studies included were from a diverse range of countries in Europe, Asia and the Americas, which allows for more generalisable conclusions. There are many differences between these geographical regions, including ethnicity profiles, socioeconomic status, maternal immunization programmes and prevalence of pertussis, tetanus and diphtheria infections. The decay rates of transplacental maternal antibodies against PRN-, PT- and PRN-specific antigens did not differ by binary World Bank income categories. Interestingly, IgG glycosylation, which can influence the rate of IgG transfer across the placenta, has been reported to differ greatly between LMICs and HICs [3], which could suggest a difference in maternal antibody decay kinetics. However, researchers have found that immunization is able to overcome these differences, producing antigen-specific IgG with comparable glycosylation profiles in people in both regions [38], which would support our finding.

## 6. Limitations

The studies were from known collaborators and not identified through systematic review as the research question addressed in our study was different to the research questions addressed by the various included studies.

Previous studies have proposed that antigen-specific maternal antibodies that have resulted from natural infection decay at a slower rate than vaccine-induced maternal antibodies [39]. This has been demonstrated mostly in studies of the live measles vaccine, including a study conducted in the UK by Brugha et al [40]. We had no data on whether mothers who were not immunised during pregnancy developed their IgG antibodies from natural infection or from previous vaccination. It is likely that tetanus antibodies were from vaccination rather than infection, similarly with diphtheria antibodies, although diphtheria does circulate in some of these countries. Pertussis antibodies would be partly vaccine-derived and partly developed following natural exposure, due to high circulation of pertussis and high prevalence of vaccination.

The studies included were from diverse regions in the world with samples processed at different laboratories, using different assays. Whilst these sources of variation may have introduced variability into the dataset, country level differences were accounted for in all models using random effects, and only within-person changes were analysed to remove the impact of laboratory differences.

## 7. Conclusion

New vaccines are in development that are likely to be authorised for administration in pregnancy, and maternal antibody interference in infant vaccine responses will continue to be one of many issues that requires careful consideration. Our findings suggest that although starting maternal antibody concentrations differ, the decay is unaffected by measured maternal or infant factors. These data can be utilised in mathematical models and to inform policy decisions and aid vaccination programme implementation in the UK.

## Supplementary Material

Supplementary material

## Figures and Tables

**Table 1:** Characteristics of included studies.

Country	Study Type	Vaccine	Gestational Age at Vaccination			Maternal Age (years)			Infant age (weeks) at second sample			Birthweight (grams)		
			N	N Miss	Median [IQR]	N	N Miss	Median [min, max]	N	N Miss	Median [min, max]	N	N Miss	Median [IQR]

Belgium [20]	Obs	None (post-partum)	0	26		26	0	31.3 [25.9, 39.5]	26	0	8.1 [3.1,8.7]	25	1	3420 [3280, 3630]
		Tdap3	48	1	28.9 [27.1, 31.1]	49	0	30.7 [20.5, 41.7]	49	0	7.9 [6.3, 10]	49	0	3300 [3120, 3706]
Canada [21]	RCT	Td	118	0	34.5 [34,35]	118	0	31 [20, 44]	118	0	9.1 [8, 11.9]	118	0	3400 [3100, 3800]
		Tdap5	116	0	35 [34,35]	116	0	31 [21,44]	116	0	9.3 [8.4, 11.6]	116	0	3500 [3100, 3800]
Guatemala	RCT	Td	129	13	26 [22.4, 30.3]	142	0	24 [18,39]	116	26	8.6 [5.1, 14.1]	135	7	2930 [2690, 3190]
		Tdap3	130	12	25.7 [21.6, 30.4]	142	0	23 [18,40]	112	30	8.7 [5.9, 13.0]	135	7	2954 [2640, 3180]
The Netherlands [22]	RCT	None (post-partum)	0	50		50	0	32.1 [24.4, 41.4]	50	0	8.7 [7.9,9.1]	50	0	3375 [3192, 3740]
		Tdap3	54	0	31.1 [30.4, 31.7]	54	0	32.4 [23.8, 42.7]	54	0	8.7 [8.1,9.7]	54	0	3430 [3094, 3764]
Pakistan [23]	Obs	None	0	223		212	11	26 [16,40]	124	99	6 [3.1,7.1]	216	7	2800 [2500, 3000]
Thailand [24]	Obs	Tdap5	235	0	30 [29,33]	234	1	29.5 [18.3, 41]	235	0	9 [7.1, 11.4]	235	0	3185 [2915, 3390]
UK1 [25]	Obs	None	0	15		0	15		15	0	7.1 [5.9,9.4]	15	0	3565 [2920, 3970]
		Tdap5-IPV or	14	2	30.5	14	2	32	16	0	7	16	0	3670
		Tdap3-IPV			[30,34]			[24,40]			[5.9, 7.7]			[3495, 3865]
UK2 [26]	RCT	Tdap3-IPV	57	0	30.1 [28.6, 31.1]	0	57		49	8	8.6 [7.6, 12.4]	57	0	3400 [3012, 3830]
		Tdap5-IPV	53	0	29.1 [28.3, 30.3]	0	53		49	4	8.7 [7.1, 10.6]	53	0	3520 [3160, 3750]
USA [27]	RCT	None	13	0	30.7 [30.6, 31.6]	13	0	26 [18,38]	13	0	8 [6,11]	0	13	
		Tdap5	30	1	31.3 [30.3, 32.3]	31	0	30 [18, 43]	31	0	8.1 [5.7, 10.3]	0	31	
Vietnam [28]	RCT	TT	0	39		39	0	25.4 [16.4, 37.8]	39	0	9.3 [2.9, 10.4]	39	0	3000 [2900, 3300]
		Tdap5	47	0	25.1 [23.2, 29.1]	47	0	25.3 [16.4, 42.3]	47	0	9.1 [4.7, 10.7]	47	0	3100 [2800, 3400]
			1044	382	30.9 [28,34]	1287	139	28.7 [16,44]	1259	167	8.7 [2.9, 14.1]	1360	66	3180 [2825, 3500]

Obs: Observational (non-randomised) study, RCT: Randomised controlled trial, N Miss: number of infants with missing data, Tdap3: Tetanus, diphtheria, 3-component acelluar pertussis (pertuss toxoid, pertactin and filamentous haemagglutinin) vaccine, Tdap5: Tetanus, diphtheria, 5-component acelluar pertussis (pertussis toxoid, pertactin, fimbriae 2& 3, and filamentous haemagglutinin) vaccine, IPV: inactivated polio vaccine, TT: Tetanus toxoid vaccine, Td: Tetanus and diphtheria containing vaccine.
