# Supplementary material for: The half-life of maternal transplacental antibodies against diphtheria, tetanus, and pertussis in infants: an individual participant data meta-analysis
Source: Vaccine. Author manuscript; Available in PMC 2022 Nov 15. (PMC9664482; doi:10.1016/j.vaccine.2021.12.007)
Supplement: Supplementary material [file NIHMS1846455-supplement-Supplementary_material.docx]

**Appendix**

Table S 1 Tdap Vaccine Composition

| **Vaccine [Manufacturer]** | **Composition** |
| --- | --- |
| Boostrix© [GlaxoSmithKline] | 5Lf tetanus toxoid, 2.5Lf diphtheria toxoid and acellular pertussis (8µg of FHA, 2.5µg pertactin, pertussis toxoid 8µg) |
| Boostrix-IPV© [GlaxoSmithKline] | 5Lf tetanus toxoid, 2.5Lf diphtheria toxoid and acellular pertussis (8µg of FHA, 2.5µg pertactin, pertussis toxoid 8µg), inactivated polio vaccine (IPV) |
| Repevax© [Sanofi Pasteur] | 2Lf (low-dose) diphtheria toxoid, 5Lf tetanus toxoid, acellular pertussis (5µg of FHA, 3µg pertactin, 5µg fimbriae 2/3, pertussis toxoid 2.5µg), IPV |
| Adacel© [Sanofi Pasteur] | 5Lf tetanus toxoid, 2Lf (low-dose) diphtheria toxoid and acellular pertussis (5µg of FHA, 3µg pertactin, 5µg fimbriae 2/3, pertussis toxoid 2.5µg) |

Figure S 1: Half-life of maternal transplacental pertussis toxin antibody in infants from mothers who received acellular pertussis vaccines during pregnancy.


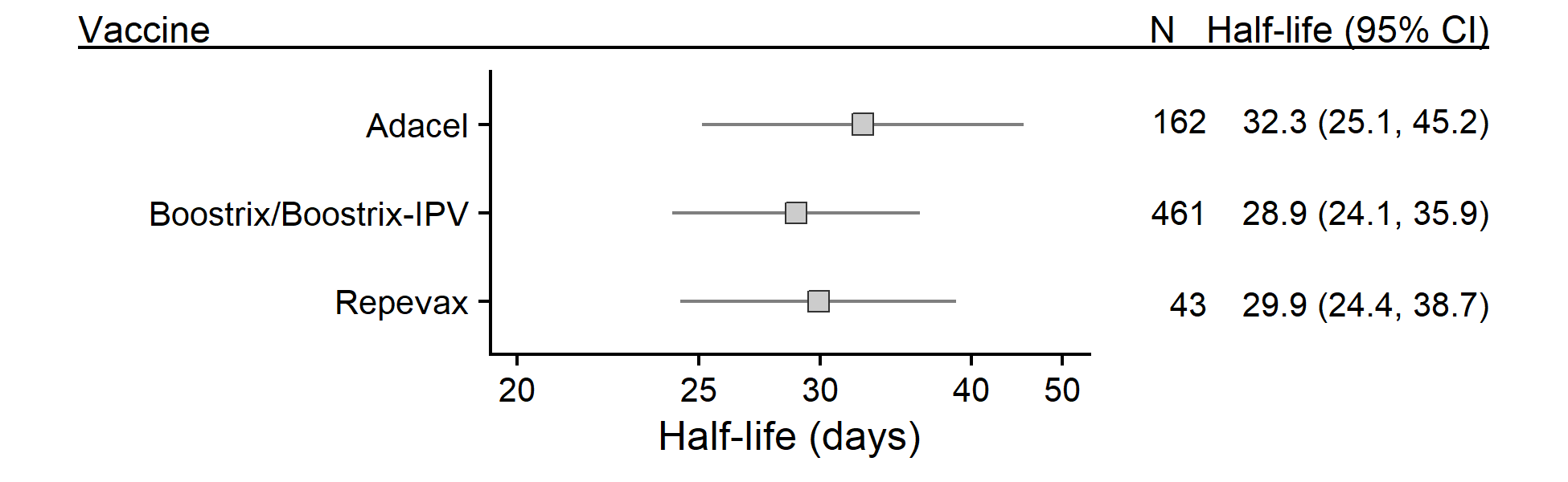


Vaccine composition detailed in Table S1.

Figure S 2: Half-lives of maternal transplacental antibody in infants, by World Bank income categories


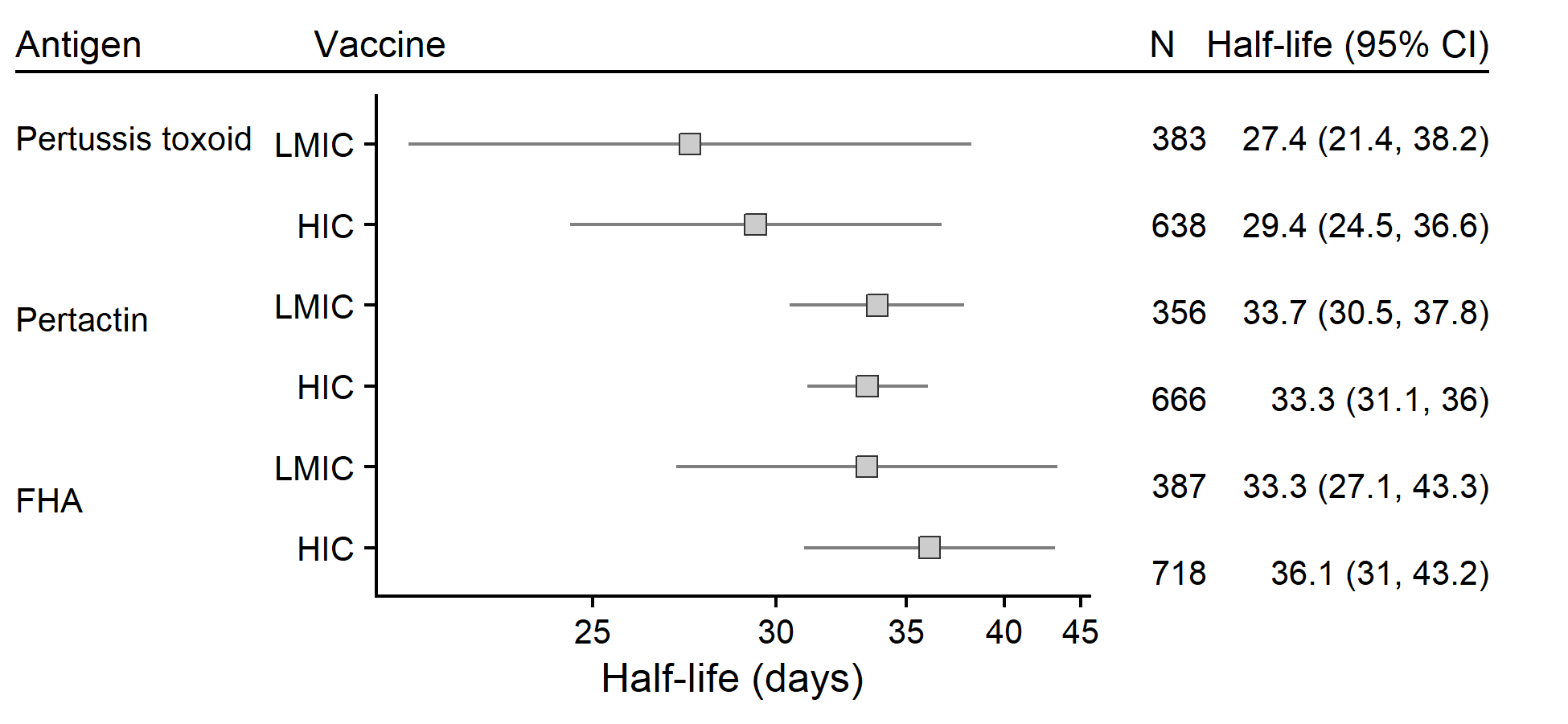


HIC: high income country, LMIC: low or middle-income country, FHA: Filamentous haemagglutinin

Figure S 3: Slope of the decay of antibody values in individual infants by study and antigen


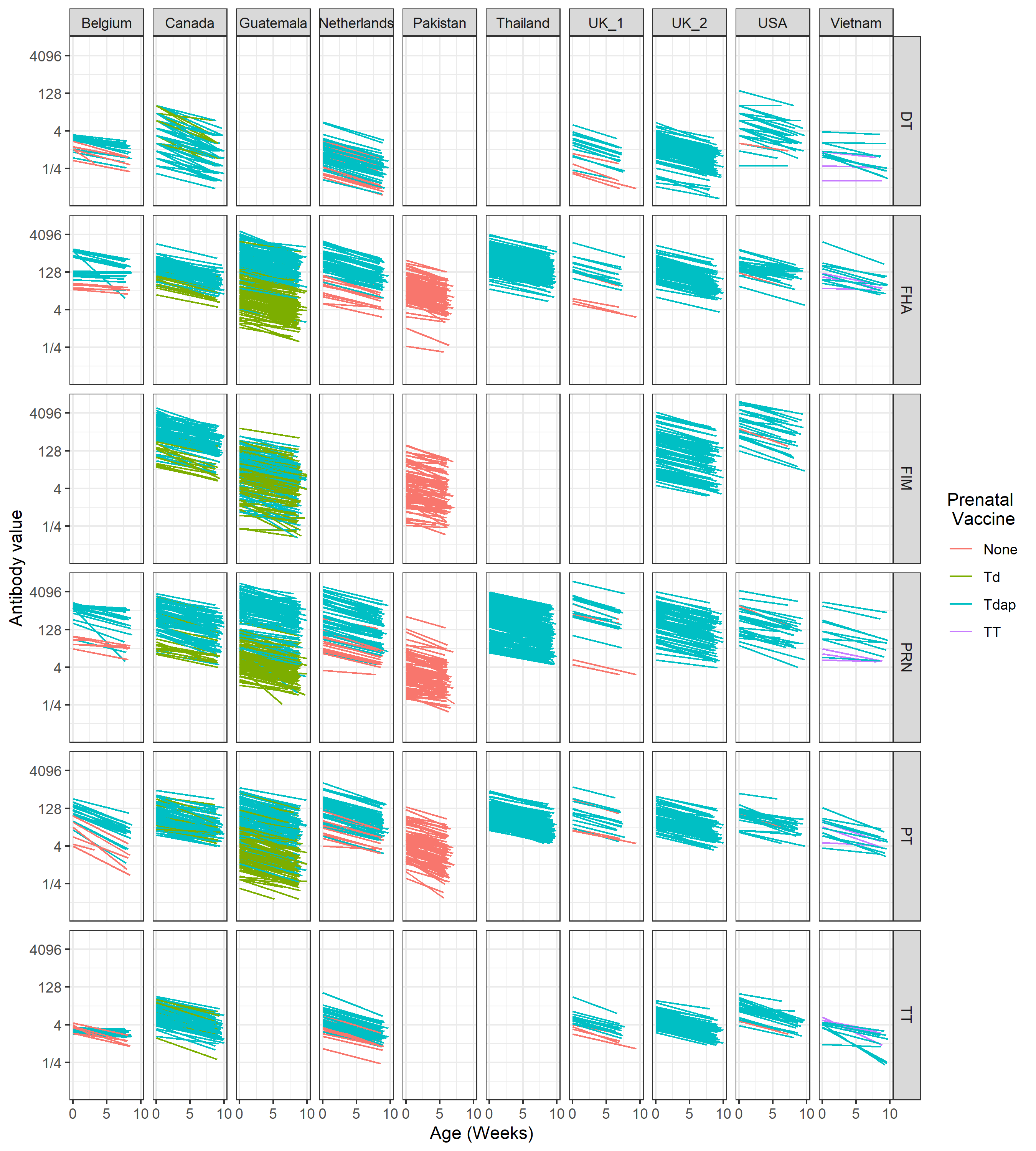


All data are presented using the same y axis, however as these data come from different studies with different assays, the magnitude of the response cannot be compared between studies. We urge the reader to consider the slope of the decay only.
